# The effects of ex vivo physical cold atmospheric plasma treatment on endothelial cells in human corneal explants and its potential for in vivo application in refractory corneal ulcers

**DOI:** 10.1038/s41598-025-18877-z

**Published:** 2025-10-08

**Authors:** Tara Brauckmann, Martin Busch, Broder Poschkamp, Lisa Lüdtke, Johanna M. Pfeil, Merlin Dähmcke, Andreas Stahl

**Affiliations:** Department of Ophthalmology, University Medicine Greifswald, Ferdinand-Sauerbruch-Strasse, 17475 Greifswald, Germany

**Keywords:** Translational research, Eye diseases

## Abstract

Physical cold atmospheric plasma (CAP) is a partially ionised, excited gas, which can be generated by applying electrical energy to a gas and comprises various biologically active components with versatile biological effects. Due to its wound-healing and antimicrobial properties, CAP gained attention for medical use. In ophthalmology, the treatment of refractory infectious corneal ulcers may represent a potential field of application. The present study aimed to investigate whether superficial CAP treatment affects deeper layers of the cornea, particularly the postmitotic endothelium, which is critical for corneal transparency and tissue integrity. Therefore, we treated the epithelial surface of human corneal explants for 2, 3, or 5 min with CAP. Before and 24 h after treatment, the corneal endothelial cells were evaluated quantitatively and qualitatively to assess endothelial vitality and functionality. Compared to control, particularly after CAP treatment, endothelial cell loss and damage was observed, indicating depth effects of CAP in corneal tissue. In this context, the treatment duration may have an impact, as endothelial cell loss tended to increase with longer exposure times. Based on these results, we used a modified treatment regimen including repetitive short CAP exposures with intermittent moistening of the cornea in an in vivo CAP application to treat patients with treatment-refractive corneal ulcer conditions. The CAP treatment had a positive outcome on corneal healing and was well tolerated.

**Supplementary Information:**

The online version contains supplementary material available at 10.1038/s41598-025-18877-z.

## Introduction

Physical plasma is a partially ionised, excited gas, which is also described as the ‘4th state of matter’^1^. In contrast to thermal plasmas, where electrons, ions, and atoms have the same amount of energy and are in a state of thermal equilibrium, in non-thermal plasmas, a few electrons carry most of the energy, while the heavier ions and atoms remain energetically ‘cold’ (< 40°C)^2,3^. In the low atmospheric pressure range, non-thermal plasma is defined as cold atmospheric plasma (CAP)^3,4^. CAP can be generated by electrically energising a neutral gas, such as helium or argon, or a gas mixture, such as air, causing the electrons to be split from the gas atoms^5^. Consequently, beside neutral gas atoms, CAP consists of excited ions and electrons, leading to the release of electromagnetic radiation, such as infrared or UV radiation as well as visible light. The interaction of these components with neighbouring air or other media secondarily stimulates the formation of reactive agents such as reactive oxygen (ROS) and nitrogen species (RNS), or free radicals. Due to these biologically active constituents, CAP has versatile biological effects on tissues and cells and gained attention for medical applications^1,6–10^.

Well described CAP effects include the inactivation of diverse microorganisms, among them various bacteria, viruses, fungi, and parasites^11–17^. Therefore, CAP treatment of surfaces, medical materials, or instruments can be used for decontamination^18–23^. Depending on the intensity and dosage, different CAP effects on human cells and tissues have been reported. Especially at high treatment intensities, apoptosis can be induced, which presents a possible therapy for susceptible tumour cells in the context of cancer research^24–27^. On the other hand, CAP treatment at lower intensities was shown to stimulate cell proliferation, migration, and tissue regeneration which in particular promotes wound healing^5,28,29^.

Due to its wound healing-inducing effects at simultaneously antimicrobial intensities and dosage, the use of CAP has great potential in dermatology. There is already evidence of its effectiveness in improving wound healing while also ensuring the elimination of microbial pathogens and tissue compatibility, which is used, for example, in the treatment of diabetic foot syndrome^13,30–34^.

In Ophthalmology, the antimicrobial and wound healing properties of CAP could be used to treat infectious, treatment-refractive corneal ulcers. Typical pathogens of these ulcers are bacteria such as *Staphylococcus aureus*, *E. coli* or *Pseudomonas aeruginosa*, as well as viruses such as *Herpes simplex* and *Varicella zoster* or fungi such as *Candida albicans*. Experimental studies have already shown that CAP treatment effectively reduces these pathogens in vitro and ex vivo in corneal explants^35–38^. Few case reports of patients with therapy-resistant corneal ulcers even demonstrated in vivo effectiveness of CAP^35^.

Although histologic examinations demonstrated structural integrity of corneal tissue with the CAP exposure used in those experiments, knowledge of in-depth effects in the cornea and, especially, the effects of CAP on corneal endothelial cells is rare. The corneal endothelial cells form a monolayer of hexagonal cells which are anchored to the overlying Descemet’s membrane via hemidesmosomes and regulate the hydration of the corneal stroma^39–41^. Therefore, the corneal endothelial cells are crucially involved in the maintenance of corneal transparency. As corneal endothelial cells are mainly in a post-mitotic state, endothelial cell loss may have sight threatening complications and should best be avoided during the treatment of corneal infectious ulcers^41,42^.

In the present study, we investigated the effects of ex vivo CAP treatment on endothelial cells in corneal explants in both a quantitative and qualitative manner to assess safety aspects on the application of CAP. We further present in vivo data from 2 patients with treatment-refractive infectious corneal ulcers, who underwent CAP treatment.

## Materials and methods

**Fig. 1:**
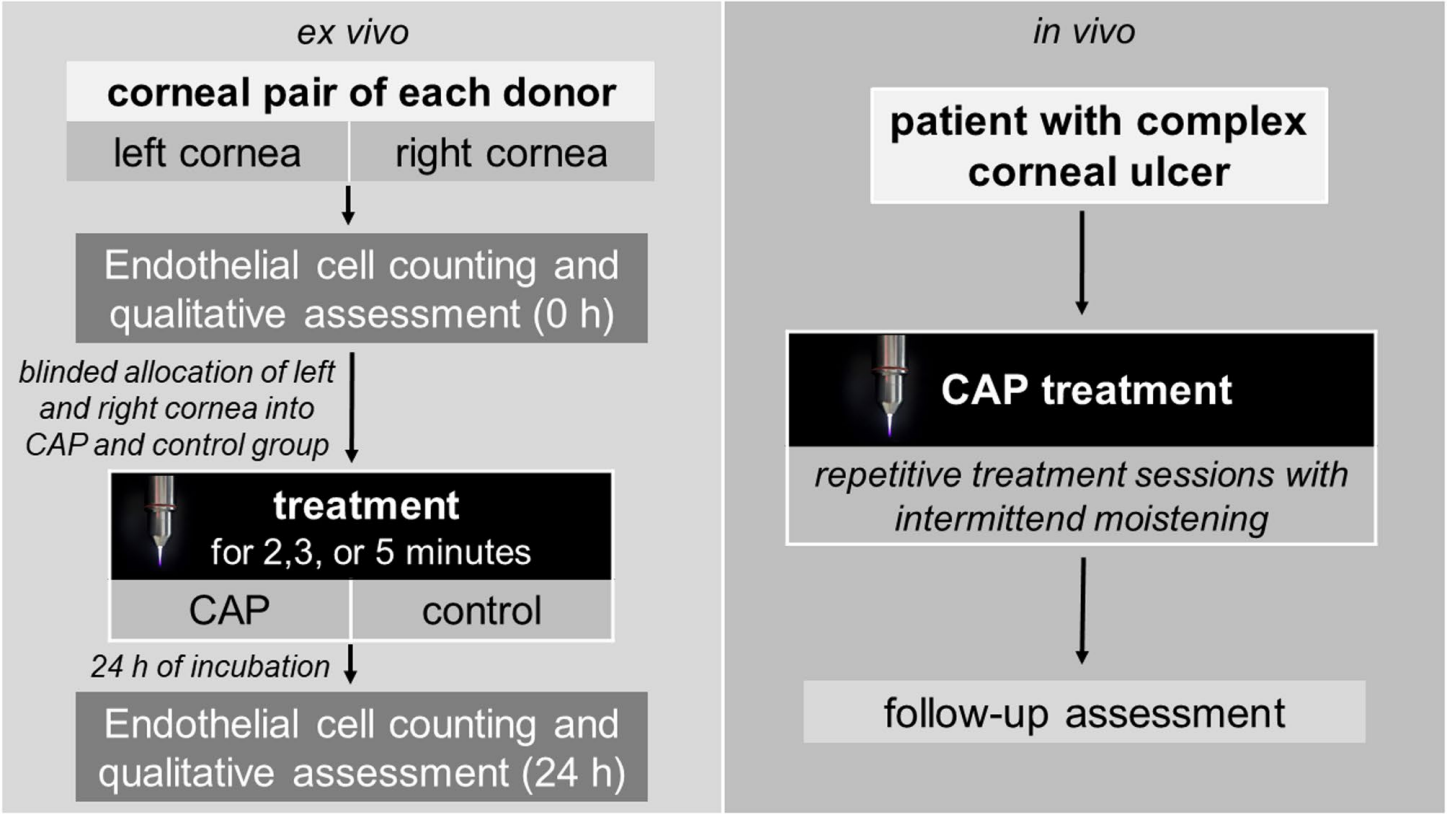
Experimental workflow of ex vivo CAP treatment of corneal explants and in vivo CAP application in patients with complex corneal ulcer conditions. The kINPen MED generates a CAP jet using argon as the feeding gas. For ex vivo control treatment, argon only was used.

With informed consent, human corneal explants were provided by the German Society for Tissue Transplantation (Deutsche Gesellschaft für Gewebetransplantation, DGFG) and obtained from the Cornea Bank at the Department of Ophthalmology at University Medicine Greifswald (Germany). Ethics approval was obtained from the Ethics Committee at University Medicine Greifswald (BB 047/20) and complied with the principles of the Declaration of Helsinki. Corneas were stored individually in culture medium for transport and storage of corneas (Culture Medium I, #P04-09701, PAN-Biotech, Aidenbach, Germany) at 37 °C. The corneas of the right and left eye of the same donor were used. Examiners were blinded to treatment conditions with one cornea being assigned to the CAP group and the other cornea of the same donor to the control group. Figure 1 illustrates the experimental workflow of our study schematically. Further details are described below.

### Ex vivo treatment of donor Corneas

For CAP treatment of the corneal explants, a prototype of the approved medical device kINPen^®^ MED (neoplas tools, Greifswald, Germany) as an indirect CAP source with argon as the feeding gas was used at a gas flow of 4.1 l/min. By applying a high-frequency voltage (2–6 kVpp, 1.1 MHz), the kINPen generates a CAP effluent visible as a CAP jet^5^. As control treatment, argon only released from the pen at a gas flow of 3.9 l/min was used.

During treatment, the corneal explant was placed in a 12-well plate containing 1 ml of culture medium to prevent the corneal tissue from drying out. CAP treatment took place at a fixed distance of the *kINPen* to the working surface (15 mm), so that the CAP jet was in direct contact with the corneal epithelium. After the treatment, the corneal explants were relocated to the culture flasks.

The corneal surface was scanned with the CAP jet by moving the 12-well plates slowly in alternating spiral and zigzag patterns to ensure consistent treatment. To investigate differences depending on the exposure time, corneas were CAP treated or control treated for 2, 3, or 5 min. After treating the corneal surface with the CAP jet facing the epithelial side, we investigated potential depth effects of CAP by assessing the corneal endothelial cells quantitatively and qualitatively.

### Measurement of endothelial cell count

24 h before endothelial cell count measurements, corneal explants were transferred to dextran-containing dehydration medium (Culture Medium II, #P04-09702, PAN-Biotech) with 2% heat-inactivated fetal calf serum (FCS). Corneal endothelial cells were counted before and 24 h after CAP or control treatment. For the microscopic cell counting procedure, the corneas were transferred into a 12-well cell culture plate filled with 3 ml of phosphate-buffered saline (PBS). Three representative images of the endothelial cell layer of each cornea were taken with an inverted cell culture microscope (Nikon Eclipse TE2000-S). The endothelial cells were counted manually from the digital images using the Robin Endothelium Analyzer (REA) analysis software (robin GmbH, Haan, Germany) and fixed frame/L method^43^. After triplicate determination of cell count, REA analysis software calculated the mean endothelial cell density per square millimeter.

### Qualitative assessment of endothelial cells

In addition to endothelial cell counting, a qualitative assessment of corneal endothelial cells was performed before (0 h) and 24 h after CAP or control treatment.

**Fig. 2:**
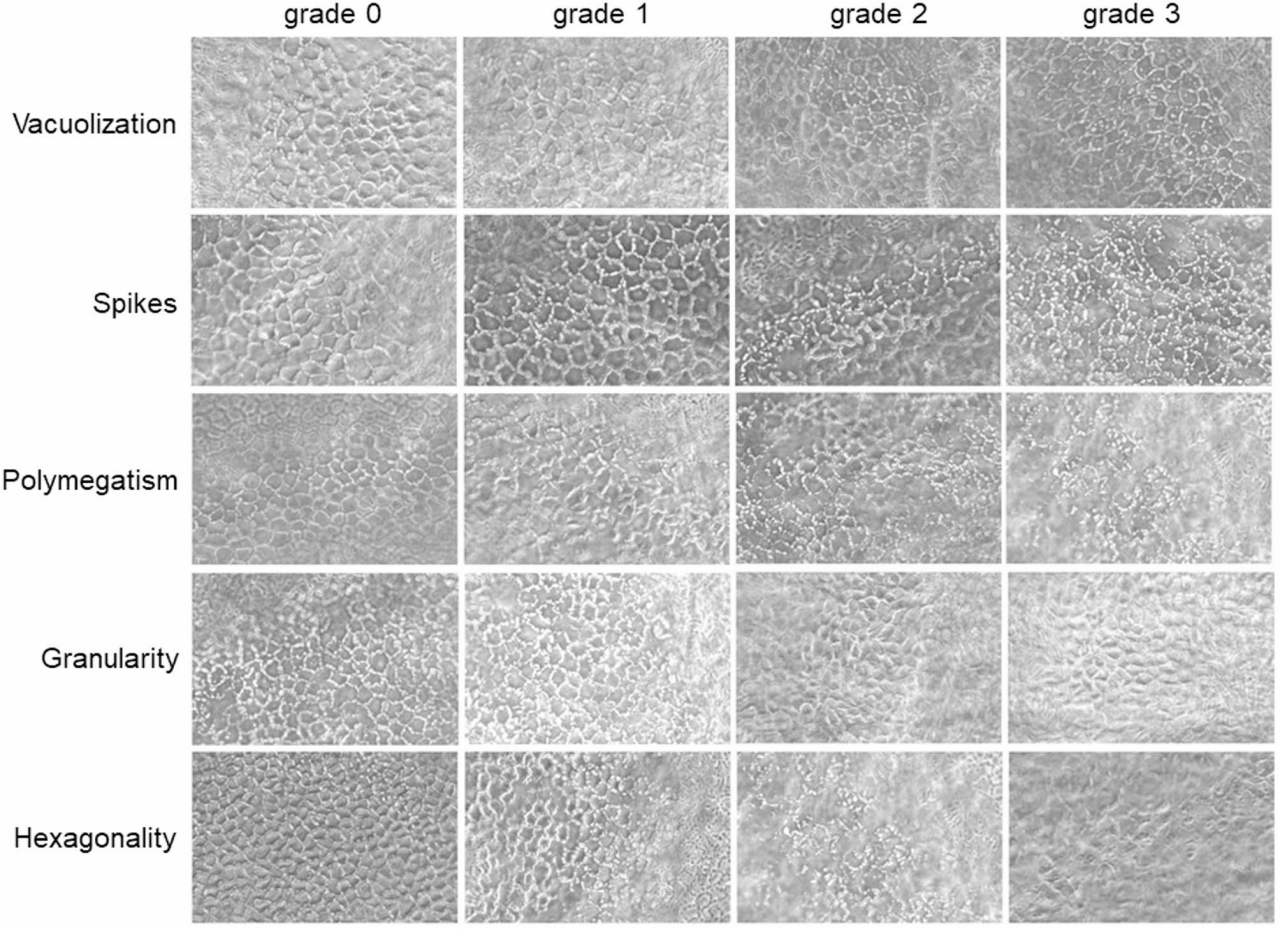
Overview of all criteria that are assessed by captured pictures. Every picture shows an example for a certain grade considering one certain criterium.

For assessing endothelial cell integrity and vitality, morphology parameters were evaluated microscopically using a grading system modified according to Hermel et al.^44^. Microscopic images were taken by blinded investigators and were graded according to the morphologic criteria vacuolization, spikes, and polymegatism on a scale from 0 to 3. For each criterion, grade 0 indicates no expression, grade 1 mild expression, grade 2 moderate expression, and grade 3 severe expression. The morphologic parameter hexagonality was assessed using the REA analysis software, which calculates the percentage of hexagonal cells for each image. This percentage was graded as follows: score 0: > 30% hexagonal cells; score 0.5: 26–30%; score 1: 21–25%; score 1.5: 16–20%; score 2: 11–15%; score 2.5: 6–10%; score 3: 0–5%. The mean score of 3 microscopic images was calculated for each cornea (Fig. 2). The criteria blur and descemet folds were assessed in relation to the entire cornea directly from the microscopic live image. Overall, a higher assessment score relates to a lower tissue integrity. The assessment of the criteria vacuolization, spikes, and polymegatism could be subsequently performed based on the microscopic images after the scoring system had been finally established, which was not possible for criteria based on microscopic live imaging. Consequently, sample sizes may vary between these criteria. Details on sample sizes are given in the respective figure legends.

### Statistical analysis

IBM SPSS Statistics V.29.0 (IBM Corporation, Armonk, NY, USA) was used to statistically analyse the data. Endothelial cell count data and scoring data were analyzed using non-parametric tests. The corneas of both eyes were treated and analyzed for each donor, with one cornea assigned to the control group and the other cornea from the same donor assigned to the CAP group. A Wilcoxon signed-rank test for matched pairs was used to compare endothelial cell loss between CAP and control at a specific treatment time, while a Kruskal-Wallis test for unpaired samples was used to compare endothelial cell loss within a treatment group between different exposure times. *p* < 0.05 was considered statistically significant. For each scoring criterium and each treatment time, a Friedman ANOVA with Bonferroni correction for 4 pairwise comparisons (CAP 0 h – CAP 24 h; Control 0 h – Control 24 h; CAP 0 h – Control 0 h; CAP 24 h – Control 24 h) was used to analyze the data. Accordingly, the level of significance was adapted to α = 0.0125.

To assess the interrater reliability, endothelial morphology parameters were graded by a second grader. Interrater agreement was assessed using Kendall’s tau c, as a non-parametric test.

The criteria blur and descemet folds, that were evaluated during live microscopy, were not assessed by the second rater. Likewise, the hexagonality was determined by the analysis software only once.

### In vivo treatment of patients with CAP

CAP treatment of patients was performed in the Department of Ophthalmology at the University Medicine Greifswald. The clinical CAP application complied with the legal and ethical principles of compassionate-use pursuant to national medical regulations (§ 21/2 of the German Medicines Act). Both patients selected for CAP treatment suffered from severe progressing sight-threatening infectious corneal ulcers and failed to respond to standard antimicrobial therapy. The decision to initiate treatment was made by the attending physicians after thorough evaluation of each case and after all conventional therapeutic options had been exhausted. Both patients received detailed information on the treatment procedure, its potential risks, and expected benefits and provided written informed consent prior to treatment, including publication of clinical images. The approved medical device kINPen^®^ MED (neoplas tools) was used for clinical CAP application.

#### Case 1: *Aspergillus fumigatus* keratitis

A 70-year-old female patient with a history of pseudophakia and type 2 diabetes mellitus presented with persistent keratitis in her left eye 3 months after cataract surgery. At initial presentation, best-corrected visual acuity (BCVA) was 1.25 (Snellen equivalent: 20/16). The ocular examination revealed conjunctival injection with temporally accentuated hyposphagma and a localized epithelial defect at 3 o’clock, surrounded by a stromal infiltrate. The remaining cornea was smooth and clear. The anterior chamber was of moderate depth, optically clear, with no cells or flare, and the iris was unremarkable, with a round, reactive, isocoric pupil. The intraocular lens (IOL) was in situ. We started an outpatient therapy with ofloxacin 5 times daily.

After 3 days without clinical improvement, the patient was admitted for inpatient treatment. On admission, slit-lamp findings showed progressive corneal involvement: the epithelial defect and surrounding stromal infiltrate at 3 o’clock had slightly increased, now reaching the paracentesis area. The anterior chamber displayed + 1 cells, and the other findings remained unchanged. Initial inpatient treatment included topical ofloxacin (Floxal EDO) and polymyxin B / neomycin / gramicidin (Polyspectran) eye drops alternating every 15 min, Povidone-iodine 1.25% 3 times daily, and hyaluronic acid gel (HyloGel) 5 times daily. Within the first 24 h, the application frequency was increased to half-hourly and hourly intervals, respectively. Microbiological testing performed from corneal swabs on admission remained negative for bacteria, Candida, and Aspergillus species.

Due to progressive worsening over the following days, including corneal thinning and the appearance of new stromal infiltrates, the anterior chamber reaction increased to flare + 2, but no hypopyon was observed. Intensive antifungal treatment was initiated: Voriconazole 1% eye drops were added on day 4 (hourly at first, then every 2 h after 12 h of use). After 5 days without sufficient clinical response, voriconazole was replaced by topical amphotericin B 0.15%. Despite maximal local antifungal treatment, the stromal ulceration continued to progress, leading to corneal thinning and infiltration into the conjunctiva and superficial sclera.

Due to the lack of clinical improvement, we introduced CAP as an adjunct treatment. After performing a deep corneal debridement under topical anesthesia, the affected areas, including the stromal ulcer and adjacent conjunctival infiltrates, were treated with a CAP pen for 3 cycles of 10 s each. Between cycles, balanced salt solution (BSS) was applied to maintain corneal hydration. Intraoperatively, visible desiccation of the ulcer base was observed. Postoperatively, the patient continued topical antifungal therapy. Microbiological testing of the debrided tissue confirmed an *Aspergillus fumigatus* infection, whereas prior swab testing had yielded negative results.

#### Case 2: complex corneal defects with amniotic transplantation

A 74-year-old female patient with a history of multiple ocular surgeries, including trabeculectomy and Ahmed valve implantation for primary open-angle glaucoma, presented with corneal decompensation (bullous keratopathy) and a corneal ulcer with infiltrations of unclear aetiology in an amblyopic eye.

At initial presentation, best-corrected visual acuity was hand movements, and intraocular pressure measured 15 mmHg. Slit-lamp examination revealed bullous keratopathy with diffuse corneal edema and bullae, a central epithelial defect (approx. 2 × 2 mm), and surrounding stromal infiltrates without hypopyon. An Ahmed Valve was visible at 12 o’clock beneath the conjunctiva. The anterior chamber was deep; however, anterior chamber flare could not be reliably assessed due to corneal edema.

Initial therapy included alternating topical ofloxacin and polymyxin B / neomycin / gramicidin (Polyspectran) eye drops every 30 min.

Two days later, the corneal infiltrate progressed into the stroma, prompting inpatient admission. Due to the uncertain infectious origin, bacterial, viral, or fungal, a broad-spectrum antimicrobial therapy was extended, including the addition of topical ganciclovir, along with intensive lubricants and ODM-5 to reduce corneal edema. Repeated microbiological testing remained negative.

After 7 days of therapy, the corneal infiltrate reduced and the patient was discharged. However, during the following 21 days, there was no improvement of the corneal ulcer and the infiltrate showed renewed progression. The patient was re-admitted, and oral aciclovir therapy was added due to a suspected viral component.

Despite intensified topical and systemic therapy, the infiltrates persisted and continued to involve the stroma.

To optimize healing conditions and reduce potential microbial load, CAP therapy was applied to the ulcer bed. Similar to our first case, we treated the cornea with the CAP pen multiple times for 5 s each, moisturising with BSS between cycles.

Following treatment, the corneal ulcer demonstrated significant improvement, with a notable reduction in infiltrations. Given the persistent epithelial defect but improved infectious status, an amniotic membrane transplantation was performed 14 days after CAP treatment.

## Results

### Endothelial cell density

**Fig. 3:**
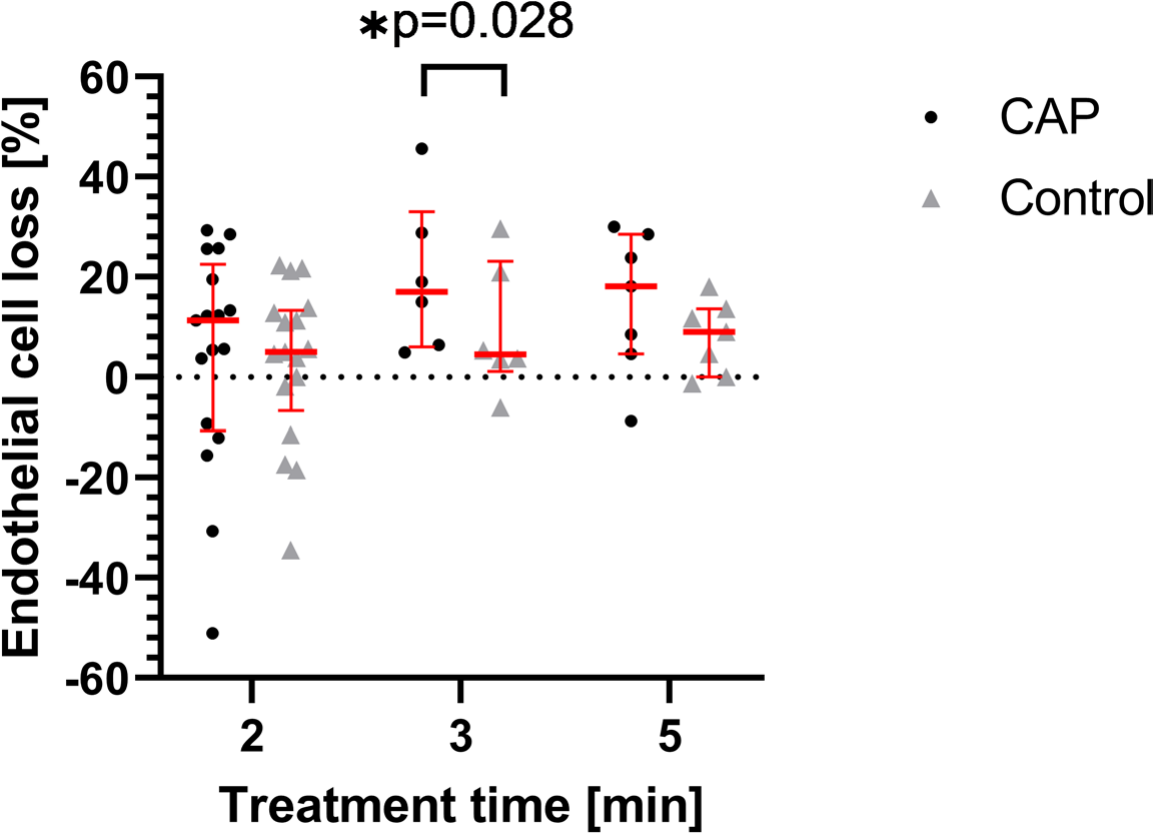
Endothelial cell loss (%) 24 h after CAP and control treatment depending on treatment time (minutes). Endothelial cell density was determined immediately before and 24 h after CAP or control treatment for 2 min. (*n* = 17 cornea pairs), 3 min. (*n* = 6), or 5 min (*n* = 7). The endothelial cell density after CAP/control treatment is expressed as percentage of the initial value before treatment (median with interquartile range, red). *Wilcoxon signed-rank test: *p* = 0.028. Note that negative values do not mean actual cell proliferation, but are due to different image areas that were selected for cell counting.

In order to investigate potential adverse effects of CAP treatment on the corneal endothelium, in a first step, we determined the endothelial cell density before and 24 h after CAP/control treatment and calculated the endothelial cell loss. Figure 3 shows the endothelial cell loss after CAP or control treatment in dependency of the treatment time. Overall, endothelial cell loss was higher after CAP than control treatment, which was statistically significant after a treatment time of 3 min (Wilcoxon signed-rank test, *p* = 0.028). There was a tendency towards an increased endothelial cell loss after longer CAP treatment times. Though not statistically significant (Kruskal-Wallis test), the highest median endothelial cell loss was observed after 5 min of CAP treatment.

### Qualitative assessment of endothelial cells

In addition to quantifying endothelial cell loss, the corneal endothelial cells were assessed qualitatively. We applied an established corneal cell scoring system, that allows conclusions to be drawn about vitality and functionality of corneal endothelial cells^44–46^. Based on microscopic images taken before (0 h) and 24 h after CAP or control treatment, the criteria vacuolization, granularity, spikes, and polymegatism were graded on a scale of 0 to 3 in a blinded fashion. The grading was carried out independently by 2 different investigators and the interrater reliability was evaluated by calculating Kendall’s tau coefficient (Table 1). According to the categorization of Landis and Koch^47^the level of agreement was in a fair (Kendall’s tau-c: 0.21–0.4) to substantial (Kendall’s tau-c: 0.61–0.8) range for the criteria polymegatism and spikes. A moderate (Kendall’s tau-c: 0.41–0.6) to substantial level of agreement was found for vacuolization. However, only a poor (Kendall’s tau-c: <0) or slight (Kendall’s tau-c: 0-0.2) level of agreement was found for the criterion granularity (Table 1), which was, therefore, excluded from further analysis.

**Table 1:** Interrater reliability.

Criteria	Kendall’s Tau-c	*p* value
Vacuolization	0.574	< 0.001
Spikes	0.424	< 0.001
Polymegatism	0.556	< 0.001
Granularity	− 0.001	0.986

Kendall’s tau-c to determine the interrater reliability. Classification <0.0 = poor, 0.0-0.2 = slight, 0.21–0.4 = fair, 0.41–0.6 = moderate, 0.61–0.8 = substantial, 0.81–1 = almost perfect^47^. *p* values < 0.001 indicate agreement between raters (Kendall’s tau-c differs significantly from 0).

**Fig. 4:**
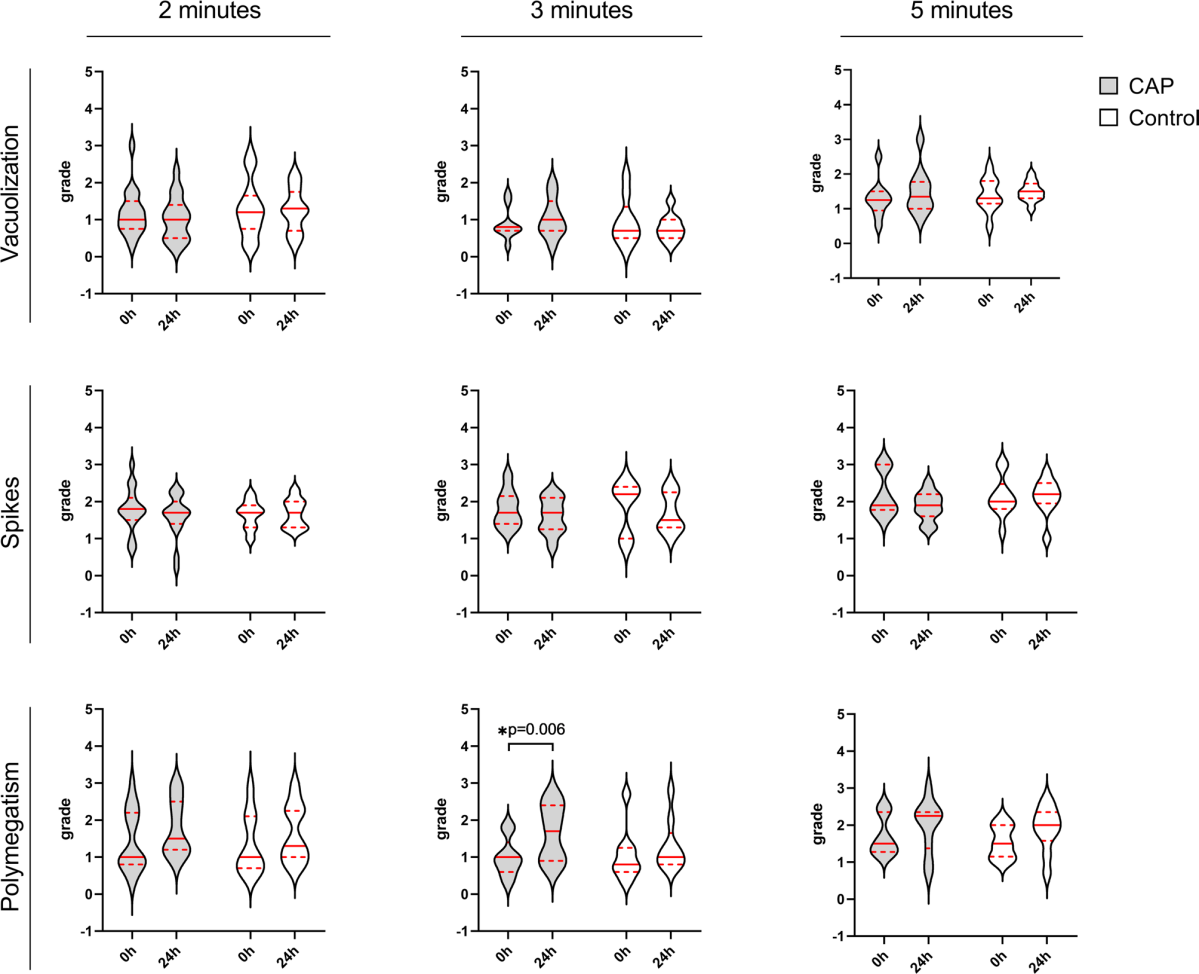
Violin plots of the endothelial cell grades for the criteria vacuolization, spikes, and polymegatism before (0 h) and 24 h after CAP or control treatment at 2, 3, and 5 min treatment time (2 min. *n* = 17, 3 min. *n* = 9, 5 min. *n* = 10). The width of the violins reflects the count of the data points at a given grade, whereby the shape of the violins reflects the distribution of data points. The horizontal bars represent the median (red solid lines) and quartiles (red dashed lines). Statistical analysis: a non-parametric Friedman ANOVA with Bonferroni correction for 4 pairwise comparisons (CAP 0 h – CAP 24 h; Control 0 h – Control 24 h; CAP 0 h – Control 0 h; CAP 24 h – Control 24 h) was calculated for each criterium at each treatment time. *Level of significance: *p* < 0.0125.

In the categories vacuolization and spikes, there were no significant score differences between CAP and control groups and there was also no difference in the score before (0 h) and after (24 h) treatment within the treatment groups (Friedman ANOVA). This was observed independently of the treatment time, indicating that CAP or control treatment had no impact on the vacuolization and manifestation of spikes in corneal endothelial cells (Fig. 4). However, a higher score of corneal endothelial cell polymegatism was observed 24 h after CAP treatment for 3 or 5 min and at a treatment time of 3 min, this effect reached the level of significance (Fig. 4).

**Fig. 5:**
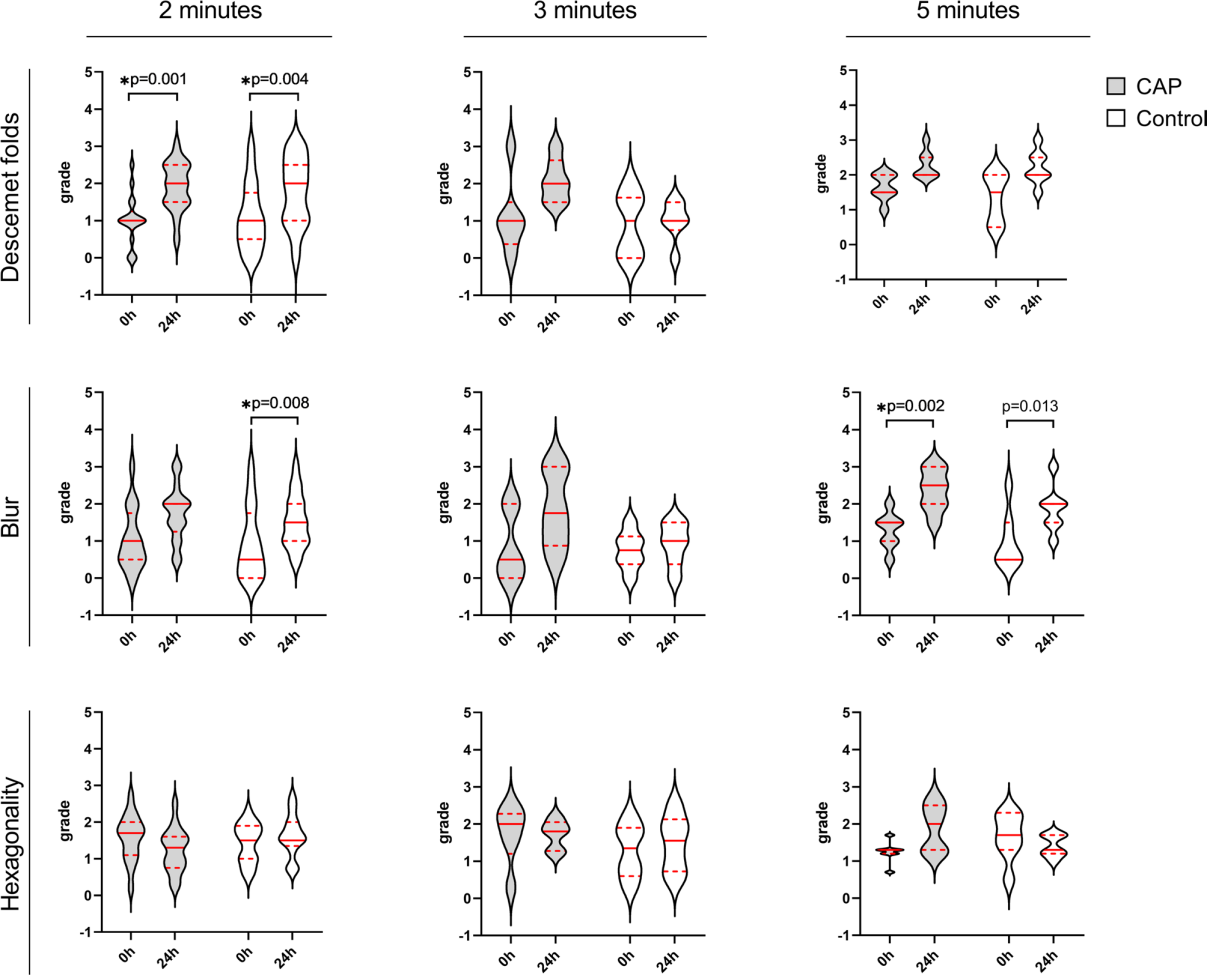
Violin plots of endothelial cell grades for the criteria descemet folds, blur, and hexagonality before (0 h) and 24 h after CAP or control treatment at 2, 3, and 5 min treatment time (2 min. *n* = 17, 3 min. *n* = 6, 5 min. *n* = 7). The width of the violins reflects the count of the data points at a given grade, whereby the shape of the violins reflects the distribution of data points. The horizontal bars represent the median (red solid lines) and quartiles (red dashed lines). Statistical analysis: a non-parametric Friedman ANOVA with Bonferroni correction for 4 pairwise comparisons (CAP 0 h – CAP 24 h; Control 0 h – Control 24 h; CAP 0 h – Control 0 h; CAP 24 h – Control 24 h) was calculated for each criterium at each treatment time. *Level of significance: *p* < 0.0125.

The qualitative criteria descemet folds and blur were graded before (0 h) and 24 h after CAP/control treatment using the microscopic live image. As shown in Fig. 5 in both the CAP and control group, the extent of descemet folds was significantly increased 24 h after treatment for 2 min compared to the baseline score before treatment (0 h). A trend to an increased extent of descemet folds was also observed after CAP treatment for 3 or 5 min. When grading the blur of the corneal endothelium before and after CAP or control treatment, an increase was predominantly observed after a prolonged treatment time (5 min) in both groups. However, even a brief CAP or control treatment lasting 2 min had an impact on endothelial blurring with a significant increase compared to the baseline value 24 h after the control treatment.

The hexagonality of corneal endothelial cells was assessed by the REA software and graded according to the percentage of hexagonal cells. An increase of the score implies a reduction of the proportion of hexagonal endothelial cells, indicating impairing effects of treatment on the corneal endothelial cell shape. At lower treatment times, CAP treatment had no influence on the grade of hexagonality. With a treatment time of 5 min, however, 24 h after CAP treatment the hexagonality grade was slightly higher than before the CAP treatment (Fig. 5).

Overall, there was a trend to a higher morphological assessment grade for the criteria polymegatism, descemet folds, and blur after CAP or control treatment, which was more pronounced in the CAP group.

### In vivo treatment with CAP

Two patients with treatment refractive infectious corneal ulcers were treated with CAP. Based on our findings from the ex vivo treatment of corneal explants, which indicate some adverse effects of CAP on the corneal endothelium and suggest that the treatment duration may also play a role in this context, the in vivo treatment regimen was adjusted. Consequently, short consecutive CAP exposure intervals with repetitive moistening of the cornea between treatments were applied. Patient history and therapeutic procedure can be found in detail in the methods section.

#### Case 1: successful treatment of Aspergillus fumigatus keratitis with CAP

**Fig. 6:**
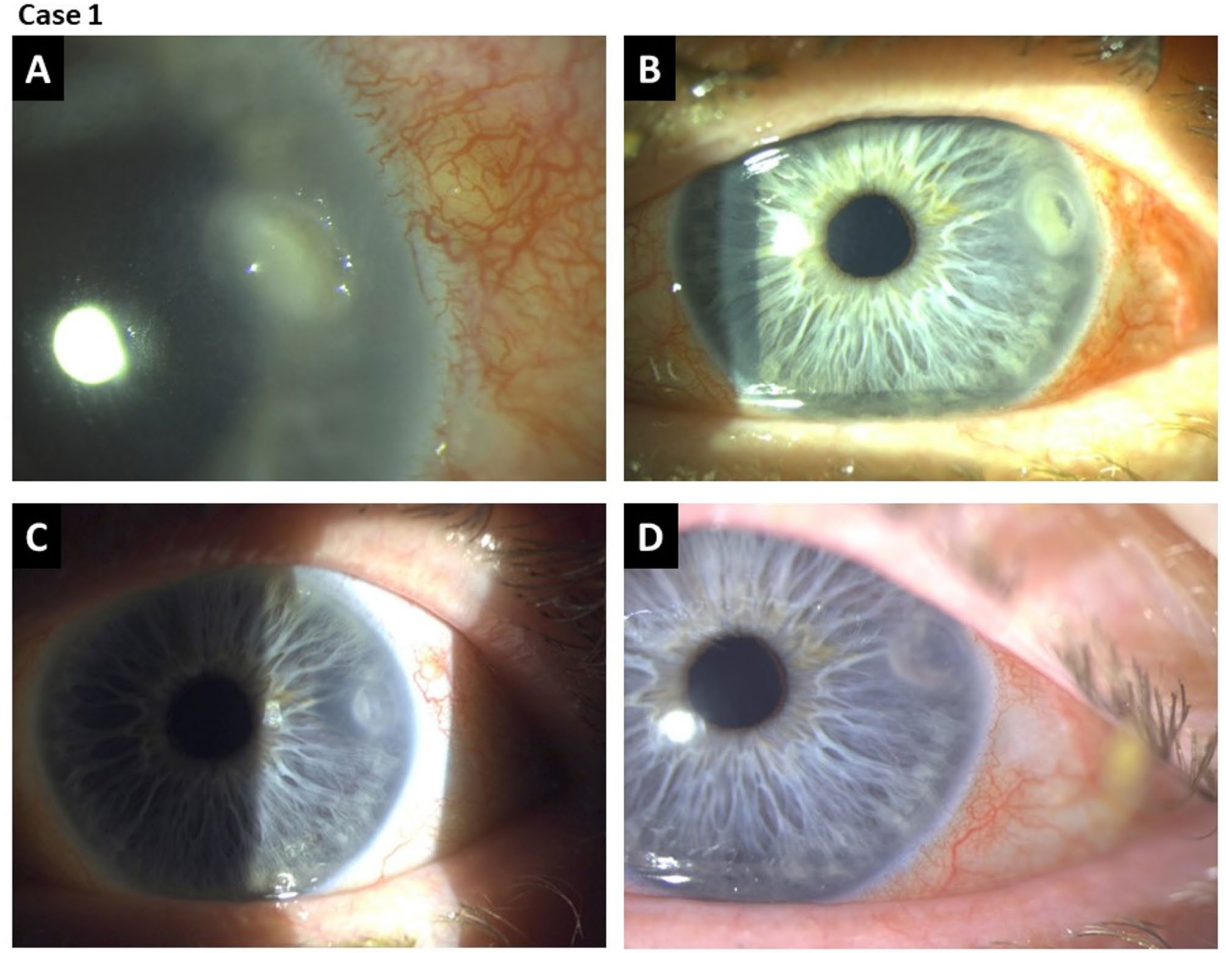
Clinical progress of *Aspergillus fumigatus* keratitis with CAP treatment. (**A**) Before treatment, (**B**) 8 hours after treatment, (**C**) 11 days after treatment, (**D**) 8 weeks after treatment.

On the first day after CAP treatment, the patient showed significant clinical improvement with rapid epithelialization and regression of the stromal infiltrate. The patient was discharged 3 days after CAP treatment with a topical therapy regimen consisting of amphotericin B eye drops every 2 hours during the day, ofloxacin (Floxal EDO) 5 times daily, HyloGel 5 times daily, and Dexamethasone EDO 3 times daily. Slit lamp follow-up examinations and fluorescein staining confirmed complete healing of the corneal defect with only minimal residual scarring (Fig. 6, Supplementary Fig. S1). Optical coherence tomography (OCT) imaging of the cornea 14 months after CAP treatment demonstrated re-epithelialization and replenishment of the stromal defect so that corneal thickness gained nearly the original extent (Supplementary Fig. S2). Furthermore, after complete corneal healing, an endothelial cell density measurement could be performed, which revealed values between 1952 and 2349 cells/mm^2^, indicating preserved endothelial integrity (Supplementary Fig. S3). Overall, no adverse effects or recurrences were observed over a 14-months follow-up period.

#### Case 2: successful treatment of complex corneal defect with CAP before amniotic membrane transplantation

**Fig. 7:**
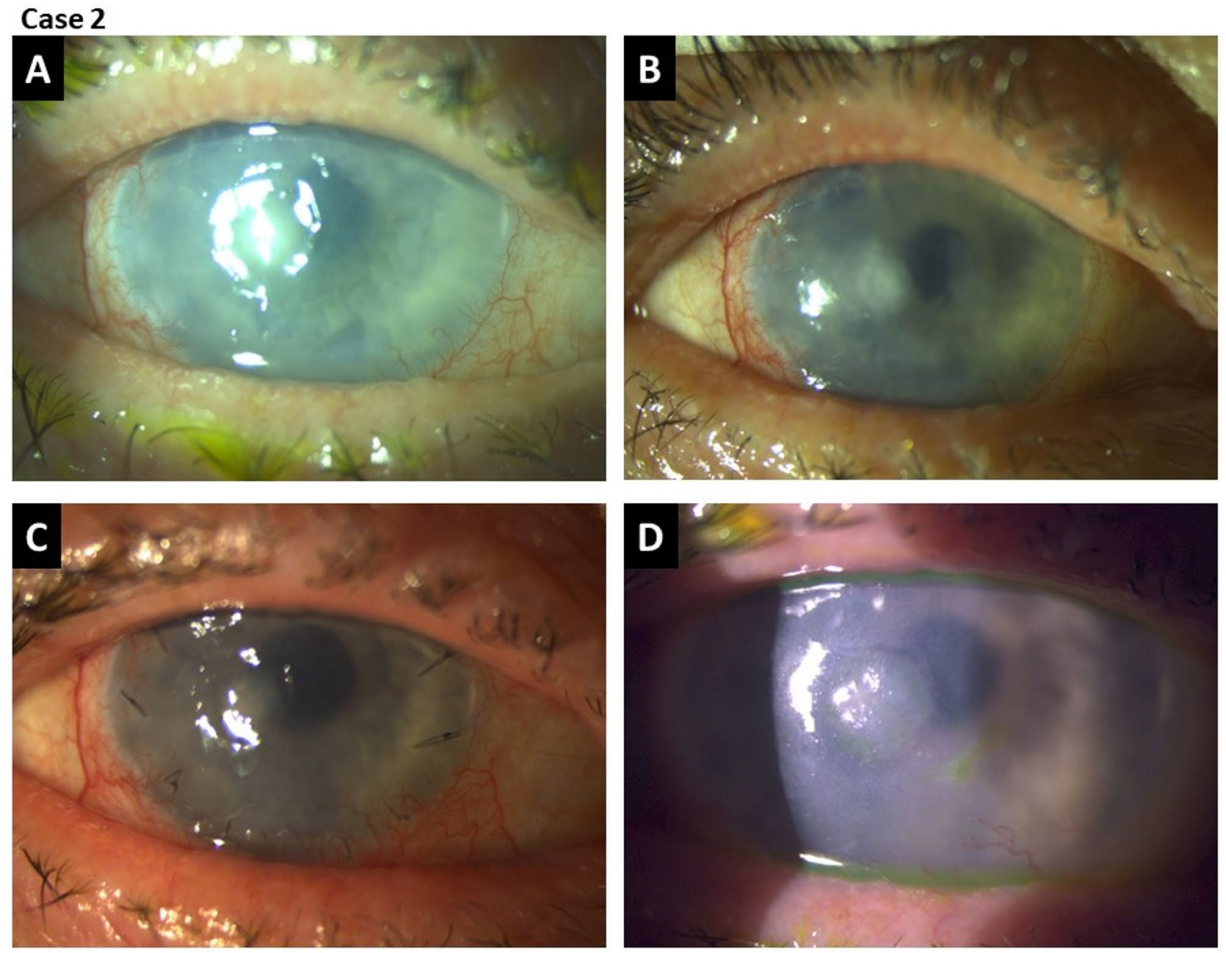
Clinical progress of a complex corneal defect after CAP treatment. (**A**) First day after CAP treatment, (**B**) 6 days after CAP treatment, (**C**) 28 days after amnion transplantation and 42 days after CAP treatment, (**D**) 42 days after amnion transplantation and 56 days after CAP treatment.

Following CAP treatment, the corneal ulcer demonstrated significant improvement, with a notable reduction in stromal infiltration (Fig. 7, Supplementary Fig. S1). Given the persistent epithelial defect but improved infectious status, an amniotic membrane transplantation was performed 14 days after CAP treatment.

One month after amnion transplantation and following complete resorption of the amniotic membrane, a small persistent epithelial defect remained. To promote final closure, serum eye drops derived from the patient’s own blood were administered 4 times a day, leading to complete epithelialization (Fig. 7).

## Discussion

In the present study, we investigated effects of CAP treatment on corneal endothelial cells after directing the CAP jet on the epithelial surface in order to asses depth effects of CAP in human corneal explants and report successful treatment of 2 patients. Corneal endothelial cells play a significant role in regulating hydration and maintaining transparency of the cornea. However, in the cornea, endothelial cells are in postmitotic state and endothelial cell loss may have sight-threatening consequences^39–41^. In accordance with a study of Lingenfelder et al., who demonstrated antibacterial depth effects of CAP in a corneal stromal tissue model^38^we observed a reduction in the corneal endothelial cell count as well as qualitative phenotypical changes that are consistent with impaired functionality^44–46^ indicating depth effects of CAP also in human corneal explants.

In this context the duration of CAP treatment may represent a critical impact factor, as we observed that corneal endothelial cell loss tended to increase with prolonged CAP treatment times. This is in line with previous reports that CAP can induce apoptosis at high treatment intensities^2,24^. Cell loss processes may explain the simultaneous increase in polymegatism, as corneal endothelium can compensate for cell loss by changing the cell structure in order to close gaps and maintain the integrity of the cell layer^48–51^. In accordance, we observed phenotypical changes of the corneal endothelium such as a higher extent of descemet folds and blur particularly after CAP treatment. Since the amount and vitality of postmitotic endothelial cells is critical for maintaining the transparency of the corneal stroma^39–41^titrating adequate CAP treatment intensity is crucial to ensure tissue compatibility.

Various research groups have investigated the antimicrobial efficacy of CAP in ex vivo corneas in dependence of the treatment time. At a treatment time of 2 min, during which CAP proved to induce the least corneal endothelial cell loss in our experiments, Reitberger et al. demonstrated that CAP was effective in eliminating *Staphylococcus aureus*^35^. Martines et al. observed effective reduction not only of *Staphylococcus aureus*, but also of *Escherichia coli* and *Pseudomonas aeruginosa*^36^ and Brun et al. demonstrated effectiveness against *Candida albicans* and *Aspergillus fumigatus* in addition to the microorganisms mentioned above^37^.

It should be emphasised that endothelial cell changes in our ex vivo experiments were also observed after control treatment. There was an increase in the expression of blurring and descemet folds. These effects may be explained by the experimental procedure. During the treatment, in CAP and control, a gas stream hits the corneal epithelium and displaces the medium surrounding the cornea, which may lead to short-term drying effects. Other experimental procedures including culture and transfer of the corneas to different media for assessment may also have an impact on corneal endothelium integrity^45,52^. Cell loss and morphological changes in endothelial cells are well-known phenomena in eye banks and frequently represent a reason why donor corneas are not approved for transplantation^52–54^. These changes can be associated with increasing culture times^52,54,55^. Moreover, it should be noted that the process of endothelial cell counting can certainly lead to variance in the endothelial cell count. We also registered negative values for endothelial cell loss, although the endothelial cells are in the post-mitotic stage and cannot proliferate. It is known that endothelial cell counts in corneal banks can be subject to large fluctuations, partly due to a lack of microscope calibration and the counting method^56,57^. For example a study of Thuret et al., who determined the deviation of manual endothelial cell counting of test corneas with known endothelial cell density, showed that more than half of 256 counts deviated more than 10% from the actual cell density^58^.

Based on our results, we adapted the treatment procedure for the in vivo CAP application in patients with treatment-refractive infectious ulcers to minimise the risk of endothelial cell impairment. Instead of a continuous treatment, we performed repetitive sessions of 5 or 10 s each. Moistening the cornea with balanced salt solution between treatments prevented the cornea from drying out. This regimen was performed once for each patient. Our treatment regimen differs in this aspect from Reitberger et al. who treated their patients continuously for 30s to a total of 4 to 6 treatment sessions with time intervals of 2 to 7 days^35^.

Despite the limitation of only 2 cases, our in vivo CAP application confirmed previous assumptions regarding therapeutical efficacy. In our first case, a refractory fungal corneal ulcer was successfully treated with CAP. The outcome and follow-up supported the antimicrobial and wound healing effects previously described^35–37^while preserving endothelial integrity (see Supplementary Fig. S3). OCT images indicated corneal tissue restoration (see Supplementary Fig. S2), which might be attributed to the reported CAP effect on extracellular matrix formation and organization^59–61^. Overall, no major tissue damaging effects were observed with our in vivo application method, neither directly after treatment nor during follow-up. This case highlights CAP as a promising adjunctive therapy for refractory fungal keratitis, potentially enhancing the antifungal efficacy of the established topical antimicrobiotic therapy and accelerating corneal healing. Reitberger et al. showed a similar effect, but with a different spectrum of pathogens. Thus, taking our data and the work of Reitberger et al.^35^ together, in vivo CAP treatment demonstrated broad antifungal effects against *Candida lusitaniae* and *Aspergillus fumigatus* in the cornea.

In our second case, we took the approach to improve the ocular surface environment, reduce microbial burden, and support corneal healing by applying CAP treatment before amniotic membrane transplantation in a case with a non-healing corneal defect. Through the release of different cytokines, amniotic membrane transplantation supports wound healing and re-epithelialization, reduces pain and has anti-inflammatory effects, making it an important part in the treatment of persistent corneal defects among other indications^62–66^. Determining the optimal timing for amniotic membrane transplantation in the presence of a potential infection remains a clinical challenge. Our data suggest that CAP can serve as a valuable tool in complex cases, particularly as a preparatory measure to sterilise corneal tissue and to enhance healing conditions before performing an amniotic membrane transplantation. In addition to the antimicrobial effects, improved wound healing, which is also a known effect of CAP^5,28,29,34^may have contributed to the successful therapy. To our knowledge, this is the first report on the potentially synergistic effects of CAP treatment and amniotic membrane transplantation.

In summary, our data confirm and complement the previous studies, whereby CAP seems to be a promising tool in the treatment of refractory corneal ulcers. With our detailed ex vivo experiments we provide a scientific basis for titration of clinical treatment times that combine efficacious treatment with endothelial cell protection. Short treatment intervals and intermittent moistening of the corneal surface ensure desired treatment effects and tissue compatibility. Further studies will be required to explore the involved wound healing mechanisms and pathways.

## Supplementary Information

Below is the link to the electronic supplementary material.

Supplementary Material 1

## Data Availability

All data relevant to the study are provided within this article or supplementary information. Further inquiries can be directed to the corresponding author.
