# Supplementary material for: The effects of ex vivo physical cold atmospheric plasma treatment on endothelial cells in human corneal explants and its potential for in vivo application in refractory corneal ulcers
Source: Sci Rep. 2025 Oct 8;15:35103. doi: 10.1038/s41598-025-18877-z (PMC12508181; doi:10.1038/s41598-025-18877-z)
Supplement: Supplementary file 1 — Supplementary Material 1 [file 41598_2025_18877_MOESM1_ESM.pdf]

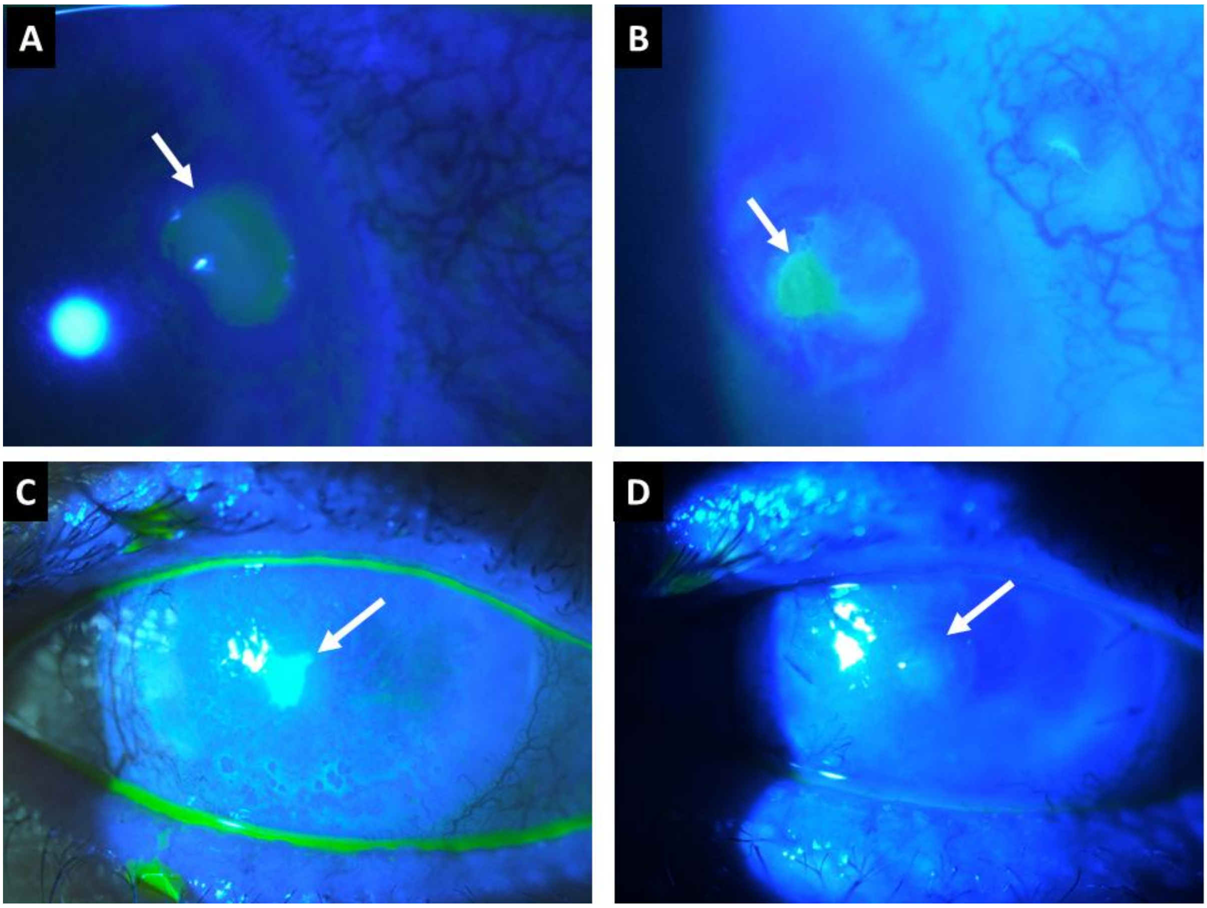

Supplementary Fig. S1. Slit lamp examinations of fluorescein-stained corneal ulcers. Following CAP treatment, slit lamp examinations revealed a substantial reduction of the fluorescein-stained ulcer area (arrows) and staining intensity. A, B: Case 1. A: before CAP treatment. B: 15 days after CAP treatment. C, D: Case 2. C: before CAP treatment. D: 6 weeks after CAP treatment.

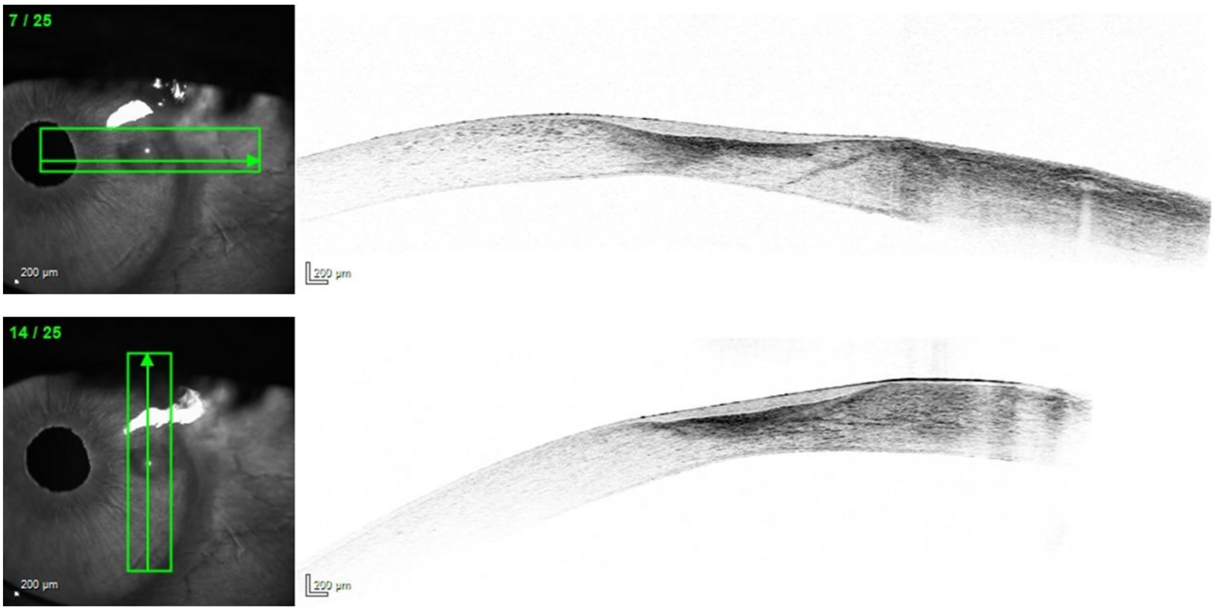

Supplementary Fig. S2. Optical coherence tomography (OCT) imaging of the cornea of clinical case 1. OCT images were taken 14 months after CAP treatment and show re-epithelialization of the cornea and covering of the hyperreflective original stroma defect with tissue, resulting in almost the original corneal thickness being achieved.

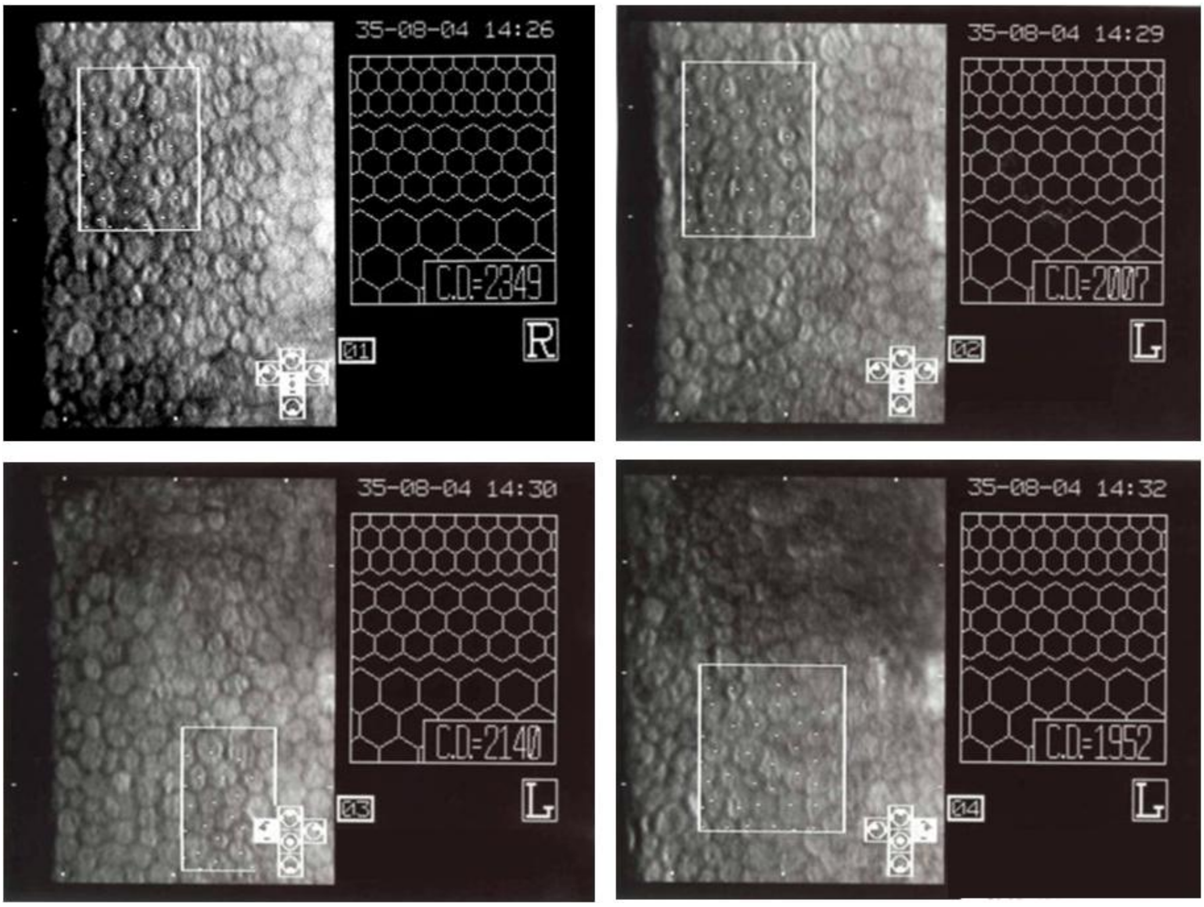

Supplementary Fig. S3. Endothelial cell density evaluation in clinical case 1. Microscopic endothelial cell density measurement and evaluation was performed 14 months after CAP treatment. Four different segments of the cornea were examined demonstrating preserved endothelial cell integrity.
